# 2639. Role of Antimicrobial Stewardship Program (ASP) in Monitoring Community Transmission of Respiratory Syncytial Virus (RSV) to Determine Optimal Timing of Inpatient Palivizumab Administration at a Pediatric Hospital

**DOI:** 10.1093/ofid/ofad500.2251

**Published:** 2023-11-27

**Authors:** Craig Shapiro, Shannon Chan, Matt Mason

**Affiliations:** Nemours Children's Hospital, Delaware, Wilmington, Delaware; Nemours Children's Hospital, Delaware, Wilmington, Delaware; Nemours Children's Hospital, Delaware, Wilmington, Delaware

## Abstract

**Background:**

The COVID-19 pandemic has impacted the transmission of RSV. Close monitoring of RSV transmission can impact optimal timing of PZ administration, as the benefit is diminished during periods of low community transmission. ASPs are in a unique position to leverage resources for monitoring epidemiology and recommending early initiation or suspension of inpatient PZ administration during periods of higher or lower RSV transmission, respectively. The role of ASPs in this capacity has not been well described.

**Figure:**
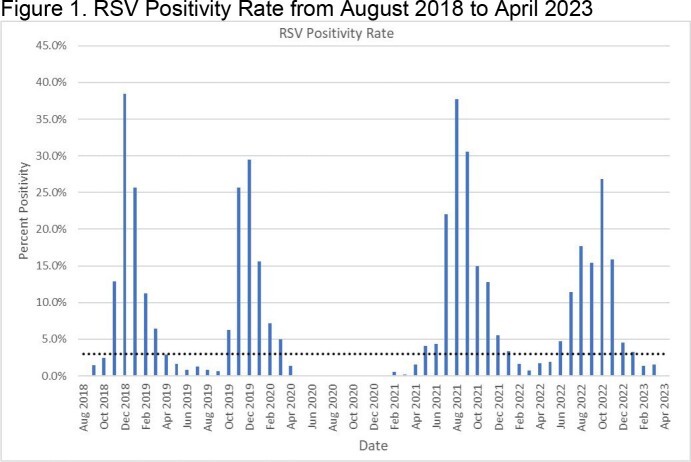

RSV positivity was determined by dividing the number of PCR tests with detectable RSV by the total number of tests performed during the specific month. Prior to the COVID-19 pandemic, inpatient palivizumab administration occurred during normal RSV seasons in the Northeast region (November to March). Prospective monitoring by the ASP was initiated in 2020 and the first suspension of inpatient palivizumab by the ASP occurred in February 2021. The dotted line represents the 3% threshold for initiation or suspension of inpatient palivizumab based on increasing or decreasing trends, respectively.

**Methods:**

In 2020, the ASP initiated prospective monitoring of respiratory viral trends using the RSV positivity rate, which was provided by the microbiology laboratory. RSV positivity rate was calculated by dividing the number of tests with detectable RSV by the total number of tests performed. Inpatient PZ administration commenced once the weekly rate exceeded 3% for two consecutive weeks, regardless of the typical RSV season (i.e., November to March in the Northeast region). Inpatient administration was suspended once the weekly rate fell below 3%, along with suppression of the PZ order set in the electronic medical record. Targeted education and communication were provided to key stakeholders for initiation and suspension of PZ ordering.

**Results:**

RSV transmission was 0% from November 2020 to January 2021. PZ administration was suspended in February 2021 and RSV positivity rate remained < 3% through April 2021 (figure 1). Administration was reinitiated in July 2021 (positivity rate 22%). The positivity rate started to decline in December 2021 and administration was suspended in February 2022 (positivity rate 1.6%). In July 2022, administration was reinitiated (positivity rate 11.4%). Administration was suspended in February 2023 (positivity rate 1.3%). The number of doses administered in each season are as follows: 52 (2018-2019), 42 (2019-2020), 14 (2020-2021), 56 (2021-2022), 65 (2022-2023).

**Conclusion:**

By monitoring local epidemiology of RSV, ASPs can provide real-time recommendations for optimal timing of inpatient PZ administration. Suspension of PZ during periods of low transmission can be considered by ASPs as a cost-saving initiative. Additional studies are needed to calculate the clinical and financial impact of this ASP intervention.

**Disclosures:**

**All Authors**: No reported disclosures

